# A ceramic liner fracture during total hip arthroplasty: a case report

**DOI:** 10.1093/jscr/rjaf346

**Published:** 2025-05-30

**Authors:** Huihui Zhou, Yan Wang

**Affiliations:** Department of Orthopaedics, The Affiliated People's Hospital of Jiangsu University, No. 8 Dianli Road, Runzhou District, Zhenjiang 212001, Jiangsu Province, China; Operating Room, Zhenjiang Hospital of Integrated Chinese and Western Medicine, No. 18 Tuanshan Road, Runzhou District, Zhenjiang 212001, Jiangsu Province, China

**Keywords:** total hip arthroplasty, ceramic fracture, operation, bilateral avascular necrosis

## Abstract

The ceramic-on-ceramic bearing has been a reliable bearing option in total hip arthroplasty (THA) for both elderly and younger patients with a long life expectancy. However, ceramic bearings are at risk of fracture. This case describes a complication during THA involving the use of a cementless press-fit acetabular shell and ceramic liner for a woman with bilateral avascular necrosis and collapse. Ceramic liner fracture occurred during its insertion owing to nonconcentric reduction. Our case report helps to expand the understanding of this rare complication of THA: ceramic liner fracture can occur intraoperatively.

## Introduction

Owing to increasing demands on quality of life, an increasing number of people with osteoarthritis (OA), whether younger or older, tend to choose total hip arthroplasty (THA), which has excellent outcomes [1]. In particular, the utilization of ceramic-on-ceramic (CoC) bearings has increased since 1972 [2]. On the basis of data from various national arthroplasty registers in 2018, CoC bearings constitute the third prevailing selected bearing surface globally [3], with greater wear resistance and biocompatibility than other materials [4]: metal-on-metal, metal-on-polyethylene, and ceramic-on-polyethylene. However, the risk of ceramic liner fracture still exists. This report presents data from a patient with a ceramic liner fracture caused by slight force during surgery. To the best of our knowledge, this complication is rarely mentioned in the literature, and how it occurs has not been described. This is the first report of a ceramic liner fracture intraoperatively.

## Case presentation

**Figure 1:**
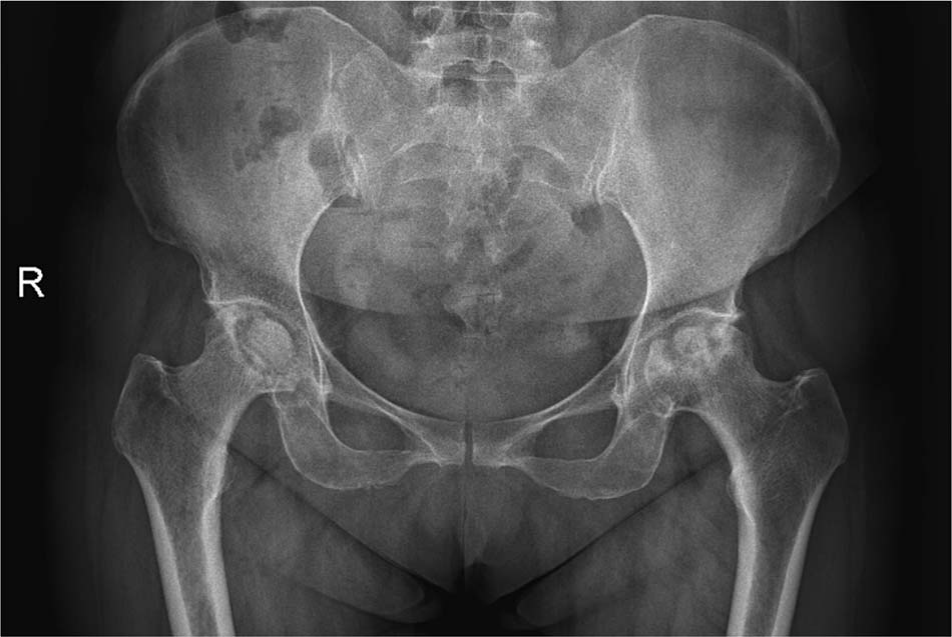
A radiograph was obtained preoperatively of a 47-year-old woman with bilateral avascular necrosis and collapse.

**Figure 2:**
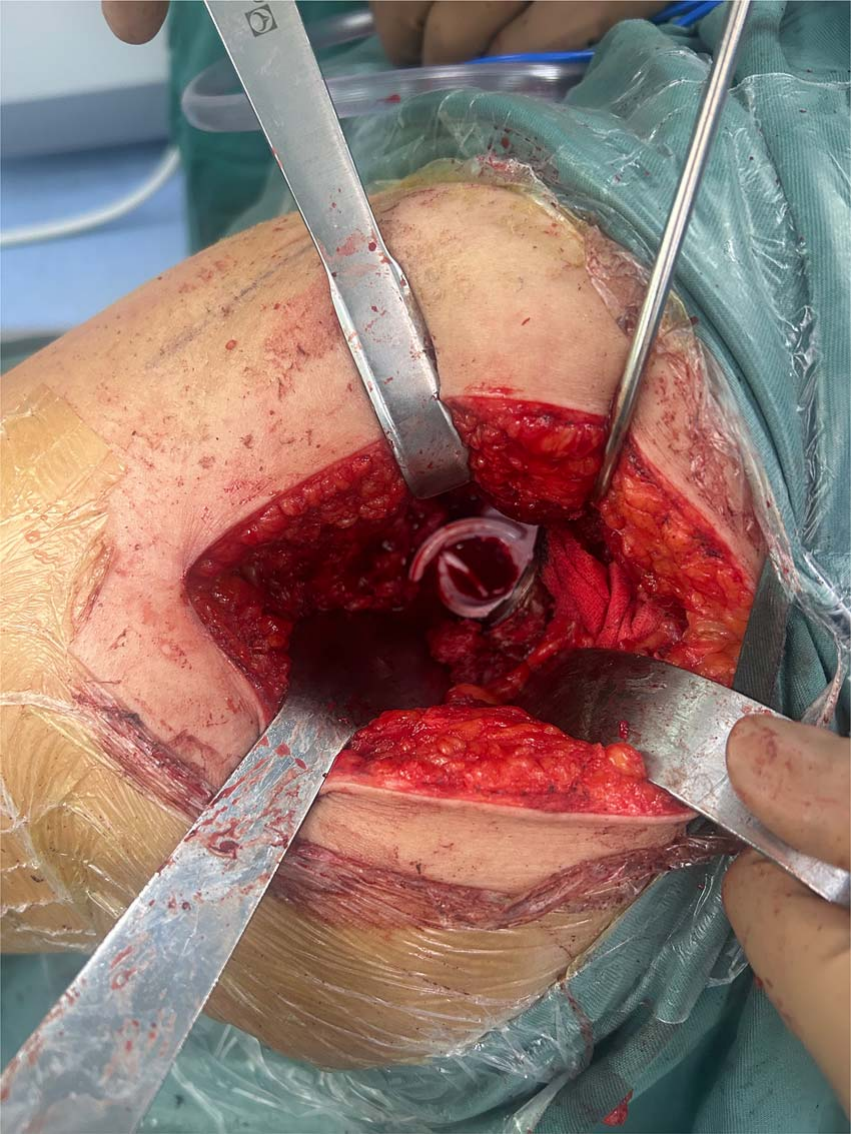
Postero-lateral approach with ceramic liner fragment.

**Figure 3:**
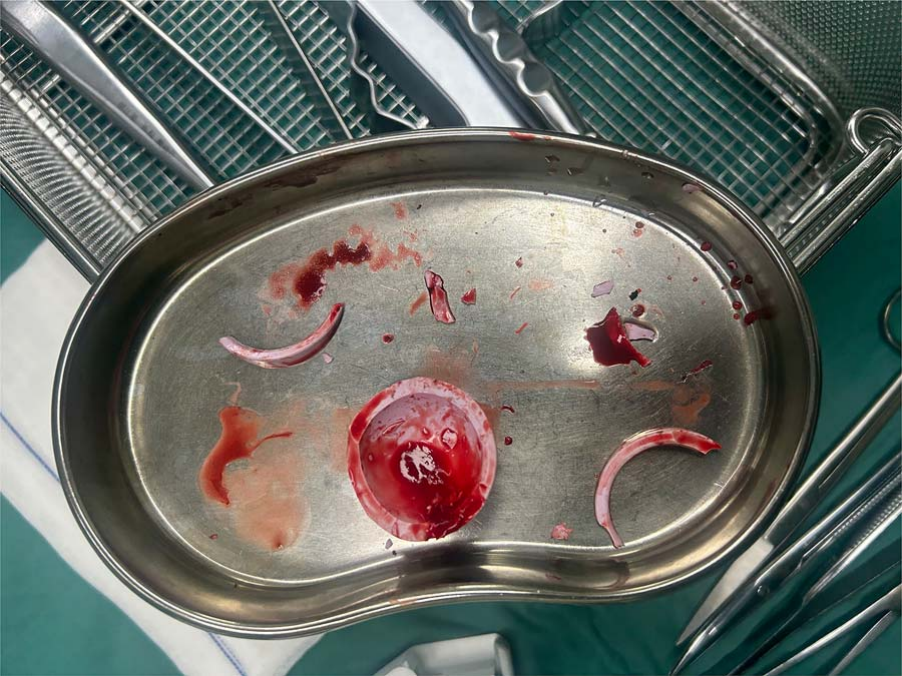
The ceramic liner fragment on the kidney basin.

**Figure 4:**
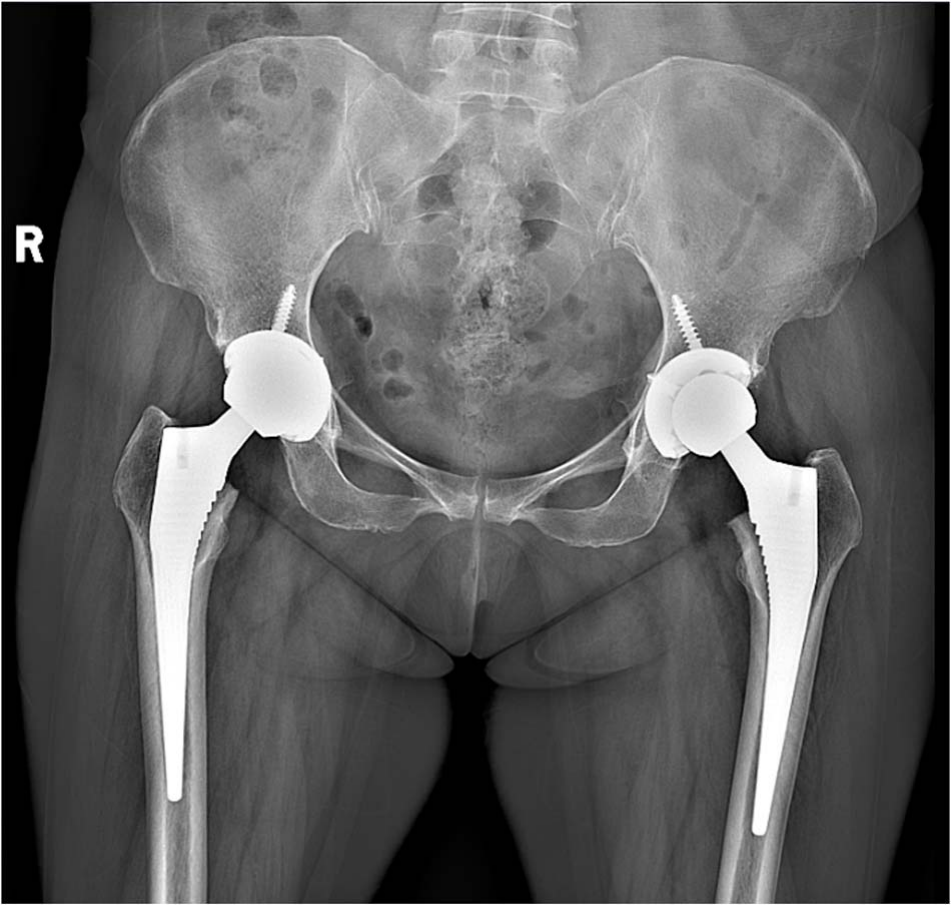
Radiograph obtained 2 months postoperatively.

A 47-year-old woman, weighing 65 kg with a height of 158 cm and a body mass index (BMI) of 26 kg/m^2^, came to our hospital with chief complaints of bilateral hip pain and difficulty walking for 1 year and underwent left THA and 1 week of right THA because of bilateral avascular necrosis and collapse. X-ray of the pelvis of both hips revealed bilateral avascular necrosis and collapse (Fig. 1). A posterolateral approach was used for the bilateral hips in the lateral decubitus position. The implant consists of a Pinnacle cup (DePuy), DELTA ceramic liner and ceramic femoral head (BIOLOX DELTA), and collarless Corail stem (DePuy). After fixation of the acetabular cup, the senior surgeon placed the ceramic liner into the metal shell by hand. Unfortunately, the liner was not placed in the centre position and became stuck. The edge of the tilted ceramic is slightly greater than the cup, perhaps only 5 mm or less. The surgeon attempted to remove the liner by tamping the edge with the plastic impactor using light force several times, but the process of doing so fractured the ceramic liner (Fig. 2). The alumina ceramic fragment of the liner was removed carefully (Fig. 3), and the site was irrigated well. A 32-mm polyethylene liner and a ceramic femoral head were implanted. Full weight-bearing was allowed on postoperative day 1 with a walking aid. The patient remained hospitalized for 8 days to monitor closely for early perioperative complications. Sutures were removed on the seventh day, and the laboratory results indicated an absence of infection. At the 2-month postoperative interval, the patient achieved a successful outcome, with complete resolution of her preoperative symptoms as well as a return to all her activities of daily living (Fig. 4).

## Discussion

Ceramic materials are increasingly being used in various surgical applications because of their excellent biocompatibility, high wear resistance, and low frictional properties [1]. However, the issue of ceramic fracture during operation has become a significant concern [5], with values between 0.004% and 0.05% for femoral heads and between 0.013% and 1.1% for acetabular liners [6, 7].

The mechanism of fracture during surgery is not known but seems to be multifactorial. The suggested risk factors for fracture are surgical technique and material composition [8]. The incidence of ceramic fractures is still closely correlated with the surgeon’s surgical technique. In the literature, ceramic liners that are not in the optimal position account for between 7.2% and 16.4% of all liners [9, 10]. Although malseated liners do not always negatively affect the success of surgery, they are more likely to fragment later than well-placed liners are. Since ceramic liner fractures frequently occur at the liner insertion site or after inappropriate surgeon impaction (a malseated liner), surgeons presemble ceramic liners in acetabular components with more than 3500 delta ceramic cases, and no liner or head fractures are reported [11]. To place the ceramic liner well, the author recommends placing the liner by hand rather than using a special tool, as they believe that a tool is not the most accurate option and that placing the liner by hand allows one to confirm the position of the liner at the same time [12]. If the ceramic liner is impacted and the position is suboptimal, then disimpaction of the ceramic pieces is very difficult, requires special instrumentation (although this instrumentation is available and can be performed successfully) and may damage the liner in such a way as in this case.

In addition, factors related to the ceramic liner itself, such as the material and impaction, also play a significant role in liner fragmentation [13]. Fourth-generation BIOLOX delta ceramic bearings were developed to reduce the number of wear fragments and improve fracture resistance, which is better than that of conventional ceramics [7]. However, cases of BIOLOX delta ceramic fragmentation with a femoral ceramic head on a polyethylene liner have been reported [14]. Ceramic fractures appear to occur when the stress exceeds the ceramic burst strength threshold, and crack initiation and propagation can occur if the stress exceeds the tensile strength of the material. The large forces required to fracture ceramic pieces are very encouraging, nearly 18 000 lb [12]. In the research of Hunger *et al*., the maximum stress was 267.5 MPa in the centre of the ceramic liner and 39.8 MPa at the liner edge [15]. Taking the stress of the ceramic into account, it becomes clear that brittleness is an important factor in the risk of fracture.

Although this patient appeared to experience linear fracture intraoperatively, it is important to acknowledge that other mechanisms may also cause implant failure.

## Conclusion

When ceramic bearings are used, fractures can occur at any time, confirming the need to carefully evaluate the position of the ceramic components before suturing the incision. An appropriate surgical plan, appropriate bearings, correct intraoperative handling of the components, and careful surgical planning are essential to achieve optimal results and reduce potential complications, including ceramic liner fractures.

## Data Availability

The datasets used and/or analysed during the current study are available from the corresponding author on reasonable request.
